# Supplementary material for: Modularity of the metabolic gene network as a prognostic biomarker for hepatocellular carcinoma
Source: Oncotarget. 2018 Feb 22;9(19):15015–26. doi: 10.18632/oncotarget.24551 (PMC5871093; doi:10.18632/oncotarget.24551)
Supplement: Supplementary file 1 [file oncotarget-09-15015-s001.pdf]

Modularity of the metabolic gene network as a prognostic biomarker for hepatocellular carcinoma

SUPPLEMENTARY MATERIALS

Downstream genes of AMPK and HIF-1

Phosphorylated AMPK (pAMPK) can activate three major transcription factors (TFs): PGC1α, CREB, and FOXO [1]. Expression of the genes downstream to these three TFs was used to quantify the activity of AMPK. HIF-1 is a transcription factor, and its downstream genes are chosen to evaluate its activity. The downstream genes chosen for AMPK and HIF-1 are listed below. The detailed procedure to obtain these genes can be found in [2].

AMPK downstream gene list (33 genes): ACADL, ACADM, ACOX1, ACSL1, ACSL5, ANGPTL4, APOC3, APOE, CAT, CPT1A, CPT2, CYP27A1, CYP4A11, CYP7A1, EHHADH, FOXA2, G6PC, GADD45A, GADD45G, HNF4A, ONECUT2, PCK1, PCK2, PDK4, TOB1, CCND2, DNMT1, G6PC3, MMP9, PRMT1, RUVBL1, ATF4, BAX.

HIF-1 downstream gene list (23 genes): ALDOA, BHLHE40, CA9, CCNB1, DDIT4, EGLN3, EPRS, ETS1, IVNS1ABP, KDM3A, MECOM, MXD1, PGK1, SERPINE1, SSRP1, STC2, TFRC, TGFB3, TMEFF1, TMEM45A, VEGFA, ALDH4A1, BNIP3.

Discrete wavelet transforms (DWT)

ITSPCA transforms the input data into the wavelet domain before denoising. This transformation step is carried out by the discrete wavelet transform (DWT). The wavelet transform decomposes signals over dilated (scaled) and translated wavelets. Compared to the Fourier transform, which decomposes signal into sine waves, the wavelet transform has a better temporal resolution. A mathematical description of DWT is [3]:

$$x[n]=\frac{1}{\sqrt{M}}\sum_k W_\phi[i,k]\phi_{i,k}[n]+\frac{1}{\sqrt{M}}\sum_{j=i}^{\infty}\sum_k W_\psi[j,k]\psi_{j,k}[n], \quad (1)$$

where $x[n]$ is the signal vector, M is the total number of points. $x[n]$, $\phi_{i,k}[n]$ and $\psi_{j,k}[n]$ are discrete functions defined in [0, M-1]. $W_\phi[i,k]$ and $W_\psi[j,k]$ are wavelet coefficients:

$$W_\phi[i,k]=\frac{1}{\sqrt{M}}\sum_n x[n]\phi_{i,k}[n], \quad (2)$$

$$W_\psi[j,k]=\frac{1}{\sqrt{M}}\sum_n x[n]\psi_{j,k}[n],\ j\ge i. \quad (3)$$

$W_\phi[i,k]$ are called approximation coefficients since they capture the low-frequency trend of the signal, and $W_\psi[j,k]$ are called detailed coefficients as they capture the high-frequency details of the signal. The $\phi_{i,k}[n]$ and $\psi_{j,k}[n]$ are wavelet basis specific to the chosen type of wavelet, e.g. Haar wavelet, symmlet, or coiflet. In general, a certain wavelet type corresponds to a specific definition of a scaling function $\phi(t)$ and a wavelet function $\psi(t)$. See Supplementary Figure 1A for an example of symmlet with 8 vanishing moments. The wavelet basis $\phi_{i,k}[n]$ and $\psi_{j,k}[n]$ can be constructed from $\phi(t)$ and $\psi(t)$ through scaling and translating. See, for example, Mallat [4].

In practice, the fast DWT algorithm obtains coefficients level by level by using filters and therefore reducing the computational complexity. This procedure passes the signal $x[n]$ through a series of filters. A tree-like diagram called filter banks can help in understanding the method (see example below). First, the signal is simultaneously decomposed using a low-pass filter g and a high-pass filter h, giving the approximation coefficients and detail coefficients of level 1. Since half of the frequencies of the signal have been removed, the filter outputs are subsequently subsampled by 2. The low-pass filter output is then passed through a new low-pass filter and a new high-pass filter but now the cut-off frequency is halved. This process goes on until it reaches the desired level of detail. Due to the nature of this decomposition, the input signal must have a dimension of 2^m, where m is a positive integer.

Here we provide an example of signal $x[n]$ with a size of 64 under a 3-level decomposition. Frequency range of the signal is 0 to f. The filter banks diagram is shown in Supplementary Figure 1B. The '$\downarrow 2$' signs means subsampling by 2. The $g[n]$ are low-pass filters and $h[n]$ are high-pass filters. DWT gives level 1–3 detail coefficients, as well as approximation coefficients obtained at level 3. Each level corresponds to information of a frequency domain, see Supplementary Table 1.

In our case, the symmlet transform was applied to the original data before a sparse PCA method was

applied. The levels and number of vanishing moments are specified in Supplementary Table 2. For an example of a symmlet transform, see Ma section 5.1 [5]. For more information about the symmlet basis, see Mallat [4].

Modularity of the metabolic gene network as a prognostic biomarker for HCC patients with no distant metastasis

To show that modularity of the metabolic gene network is predictive of patients' prognosis independent of metastasis status, we analyzed the association of modularity for 'M0' HCC patients having no spread of tumor to other parts of the body with metabolism phenotypes, stage I-IV, and tumor recurrence. The sample size of 'M0' HCC patients is 266. We analyzed the data of these 266 HCC patients using the same methods as described in the 'Materials and Methods' section.

We observed a modular gene expression pattern of the metabolic genes for these 266 HCC patients, Supplementary Figure 5A. The community structure for these 'M0' HCC patients, Supplementary Figure 5B, identified by the Newman algorithm is the same as that for all the HCC patients, Figure 1C. The association of modularity with metabolism phenotypes, Supplementary Figure 6, tumor stages I or II–IV, Supplementary Figure 7, and recurrence, Supplementary Figure 8, is consistent with the results for all HCC patients analyzed in the main text. In summary, modularity is a strong predictor, independent of metastatic status, for HCC patients.

Supplementary Table 1: DWT frequency domain of an example of signal $x[n]$ with a size of 64 under a 3-level decomposition

Level	Frequency range	Size of sample
1	$f/2$ to f	32
2	$f/4$ to $f/2$	16
3	$f/8$ to $f/4$	8
3	0 to $f/8$	8

Frequency range of the signal is 0 to f.

Supplementary Table 2: Input parameters of the ITSPCA algorithm

Parameter	Value
Number of significant leading eigenvectors n	10
Wavelet basis to be used	Symmlet
Coarsest level in wavelet transform L	4
Parameter describing the support length and vanishing moments of the selected wavelet basis, *par*	8
Adjustable constant in the diagonal thresholding step,	3
Adjustable constant in the iterative thresholding steps, β, which is directly related to level sparsity in cleaned eigenvectors.	1.5
Thresholding rule, *itthres*.	hard

REFERENCES

1. Cantó C, Auwerx J. AMP-activated protein kinase and its downstream transcriptional pathways. Cell Mol Life Sci. 2010; 67:3407–23. https://doi.org/10.1007/s00018-010-0454-z.
2. Yu L, Lu M, Jia D, Ma J, Ben-Jacob E, Levine H, Kaipparettu BA, Onuchic JN. Modeling the Genetic Regulation of Cancer Metabolism: Interplay between Glycolysis and Oxidative Phosphorylation. Cancer Res. 2017; 77:1564–74. https://doi.org/10.1158/0008-5472.CAN-16-2074.
3. Chun-Lin L. A tutorial of the wavelet transform. National Taiwan University. 2010.
4. Mallet S. A wavelet tour of signal processing the sparse way. Burlington: Elsevier Science. Third Edition. 2009.
5. Ma Z. Sparse principal component analysis and iterative thresholding. Ann Stat. 2013; 41:772–801. https://doi.org/10.1214/13-AOS1097.

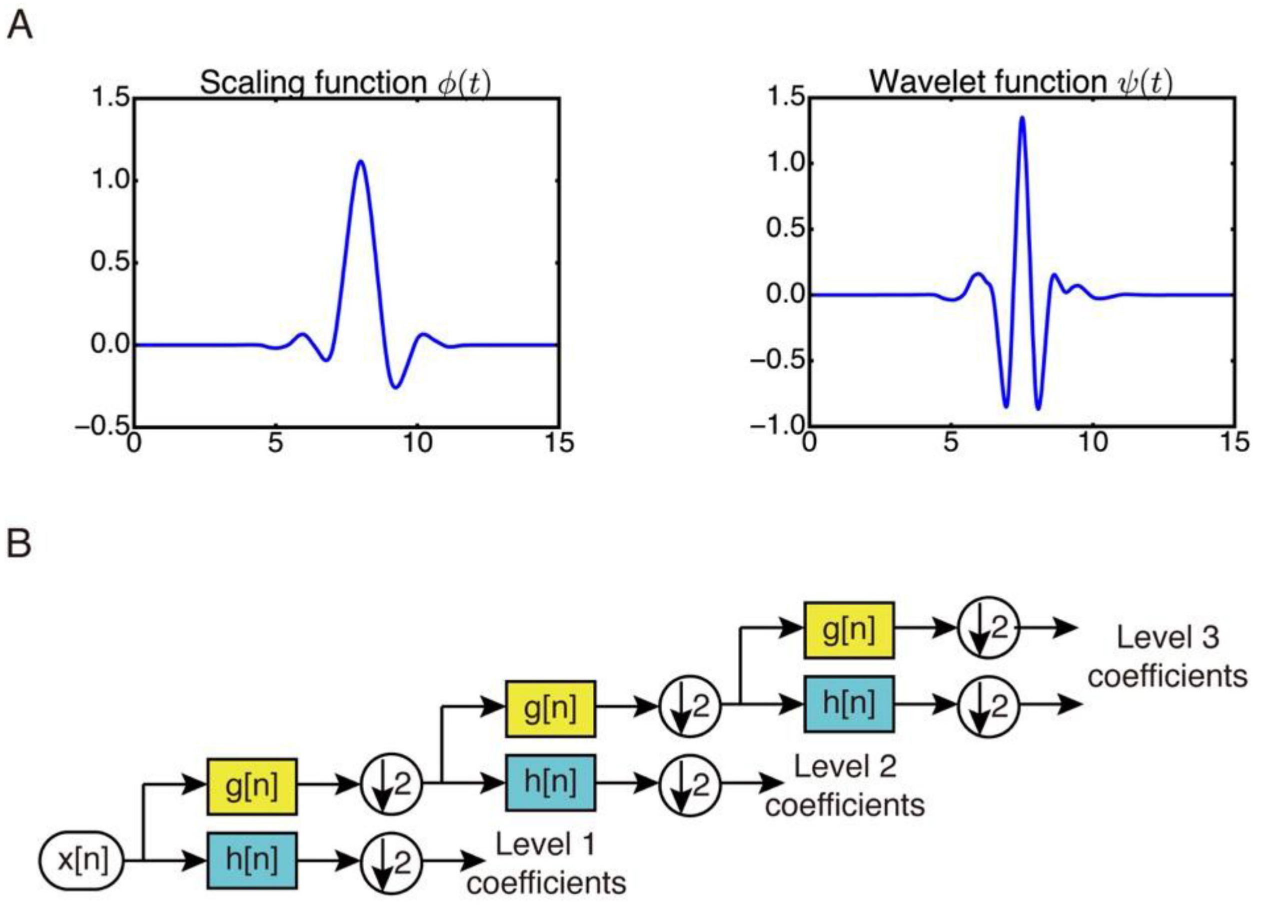

Supplementary Figure 1: Illustration of DWT. (**A**) Scaling function $\phi(t)$ and wavelet function $\psi(t)$ of symmlet with 8 vanishing moments. (**B**) Filter banks of a 3-level decomposition of signal $x[n]$. The '$\downarrow 2$' signs mean subsampling by 2. $g[n]$ are low-pass filters and $h[n]$ are high-pass filters. DWT gives level 1-3 detail coefficients, as well as approximation coefficients obtained at level 3.

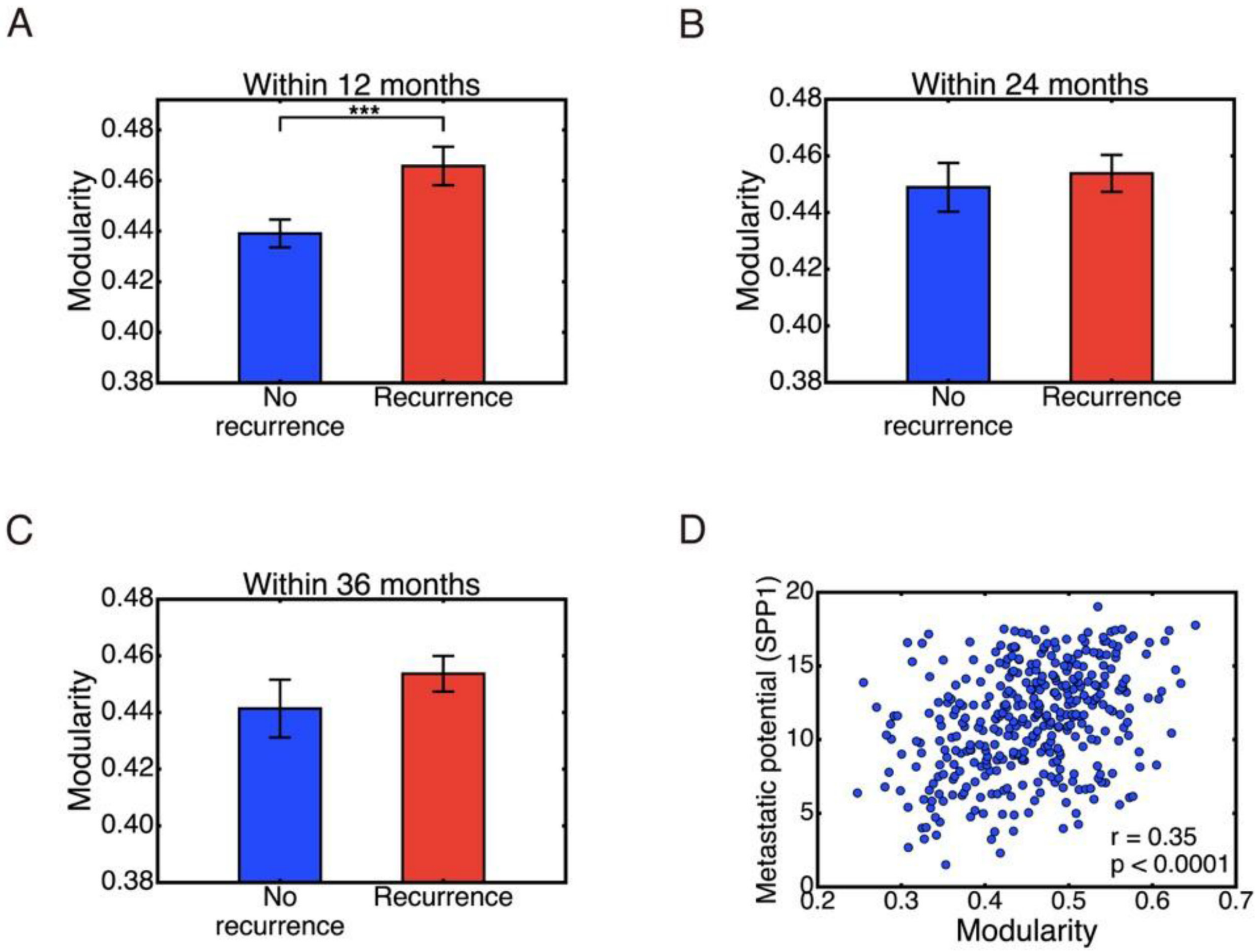

Supplementary Figure 2: Individual modularity regarding recurrence and SPP1 expression. (**A–D**) Averaged individual modularity in the recurrence group and the non-recurrence group. **(D)** Individual modularity was positively correlated with SPP1 expression (Pearson correlation, $r = 0.35$, $p < 0.0001$). Here, '***' represents. $p \leq 0.001$. If there is no labeling of the significance level, it means the difference is not significant.

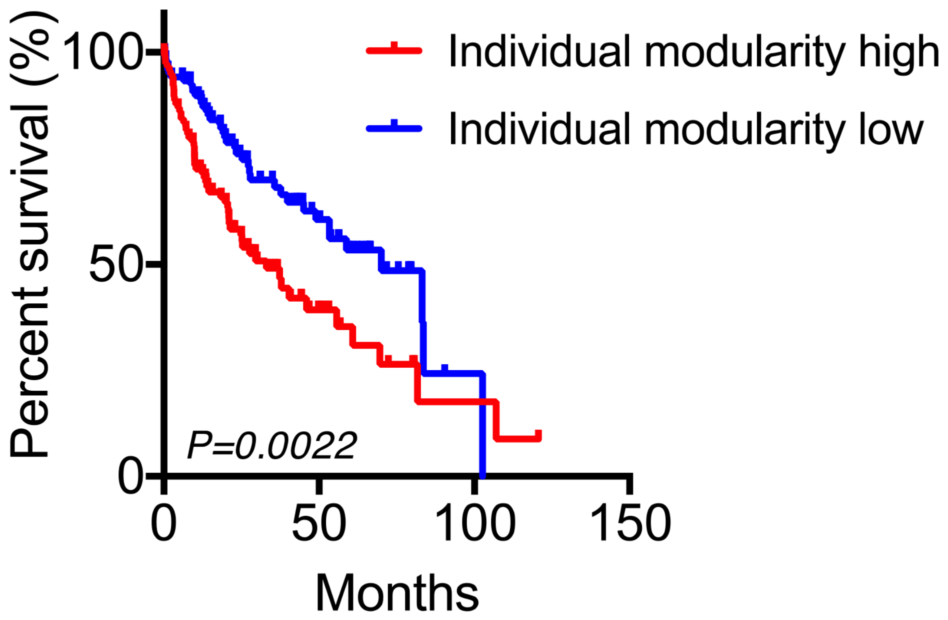

Supplementary Figure 3: Kaplan-Meier overall survival curves of HCC patients with low and high individual modularity. We chose 123 patients with the lowest individual modularity as the ‘low’ group, and the 123 patients with the highest individual modularity as the ‘high’ group.

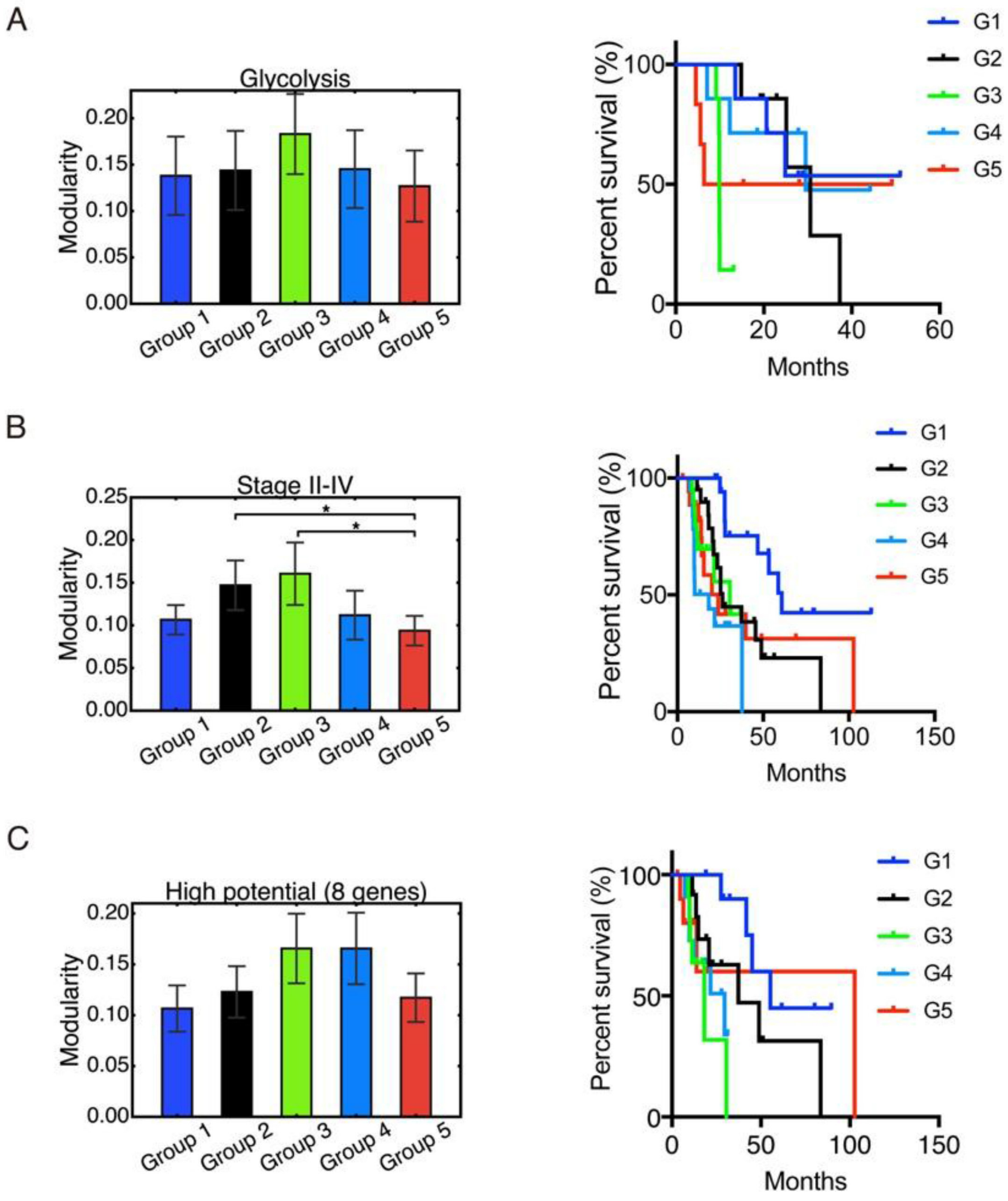

Supplementary Figure 4: Non-monotonic change of group modularity with tumor recurrence time among HCC patients with recurred tumor. (**A**) Glycolysis group. There were 37 patients that recurred, and 35 of them were selected and divided into five subgroups. Patients in G1 has the longest recurrence time, and patients in G5 has the shortest recurrence time. Recurrence time ranged from 28.9 – 9.1 months for the first three groups. Significant *p*-values among the 5 groups are as follows: p(G1,G3) < 0.01, p(G2,G3) < 0.01, and p(G3,G4) < 0.05. (**B**) Stage II-IV group. There are 100 patients with recurred tumors, and all of them were selected and divided into five subgroups. Recurrence time ranged from 55.1 – 6.4 months for the first three groups. Significant *p*-values among the 5 groups are as follows: p(G1,G2) < 0.01, p(G1,G3) < 0.05, p(G1,G4) < 0.001, and p(G1,G5) < 0.05. (**C**) High metastatic potential group. There were 57 patients that recurred, and 55 of them were selected and divided into five subgroups. Recurrence time ranged from 70.1–7.9 months for the first three groups. Significant *p*-values among the 5 groups are as follows: p(G1,G3) < 0.01, p(G1,G4) < 0.05, and p(G2,G3) < 0.05. In all cases, we find the correlation of larger modularity with worse prognosis for roughly ~60% (the top 3 groups) of patients. The correlation reversed at a recurrence time of ~8 months. Here, '*' represents $0.01 < p \leq 0.05$. If there is no labeling of the significance level, it means the difference is not significant.

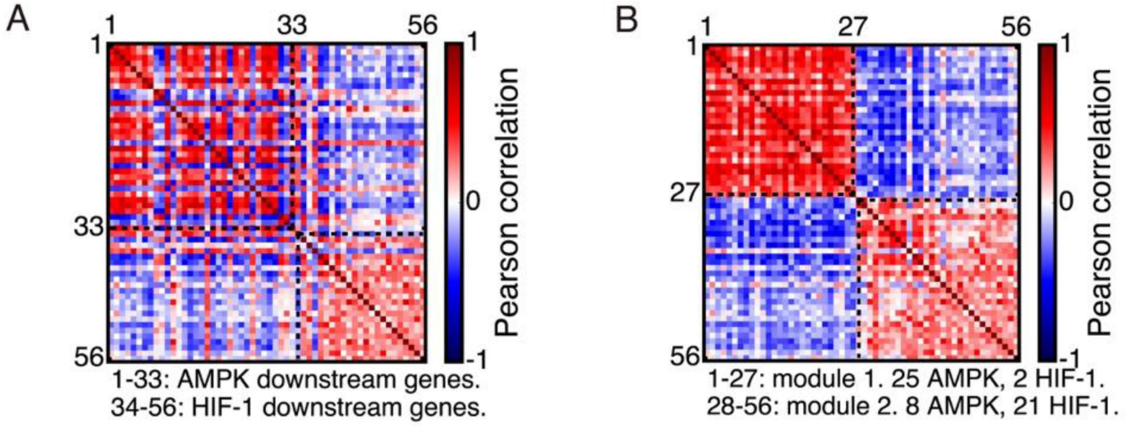

Supplementary Figure 5: Modular expression pattern of the metabolic genes for HCC patients with no distant metastasis. **(A)** Gene expression correlation matrix of the 33 AMPK-downstream genes and 23 HIF-1-downstream genes. **(B)** Rearranged correlation matrix calculated by the Newman algorithm. Two modules were identified. One module contains mainly AMPK-downstream genes, and the other contains mainly HIF-1 downstream genes. The red dashed lines along the diagonal in (A) and (B) correspond to the correlation coefficient of 1, which is the expression correlation of a gene with itself. In modularity calculation, the diagonal elements were set to 0, as it was assumed that there were no self-loops.

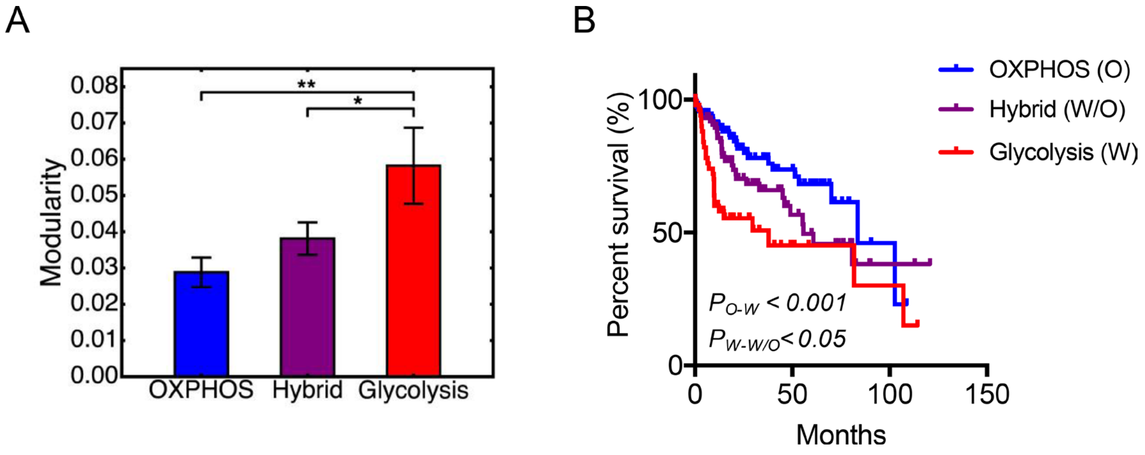

Supplementary Figure 6: Modularity and metabolism phenotypes for HCC patients with no distant metastasis. (**A**) Group modularity of three metabolism phenotypes. Here, ‘*’ represents $0.01< p \leq 0.05$, and ‘**’ represents $0.01< p \leq 0.01$. If there is no labeling of the significance level, it means the difference is not significant. (**B**) Kaplan-Meier overall survival curves for HCC patients in three metabolism phenotypes.

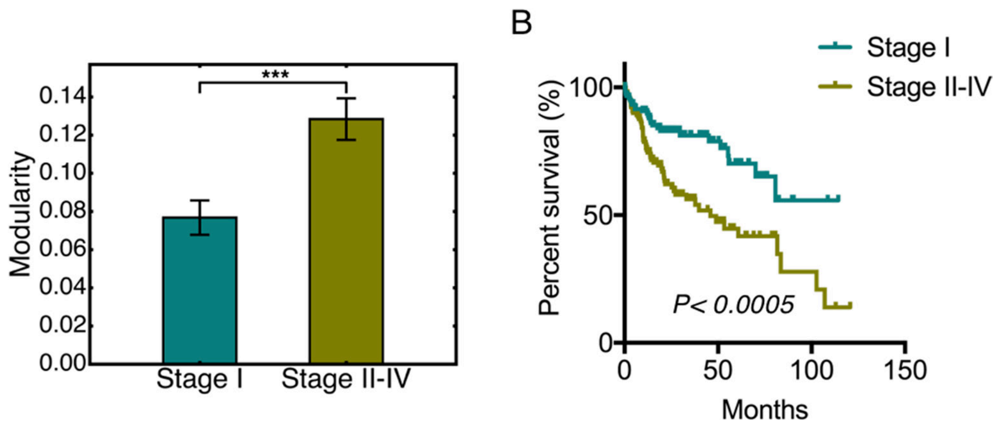

Supplementary Figure 7: Modularity and tumor stages for HCC patients with no distant metastasis. (**A**) Bar plot of the modularity for HCC patients at stage I and stage II–IV. Here, '***' represents $p \leq 0.001$. (**B**) Kaplan-Meier overall survival curves for HCC patients at stage I and stage II–IV.

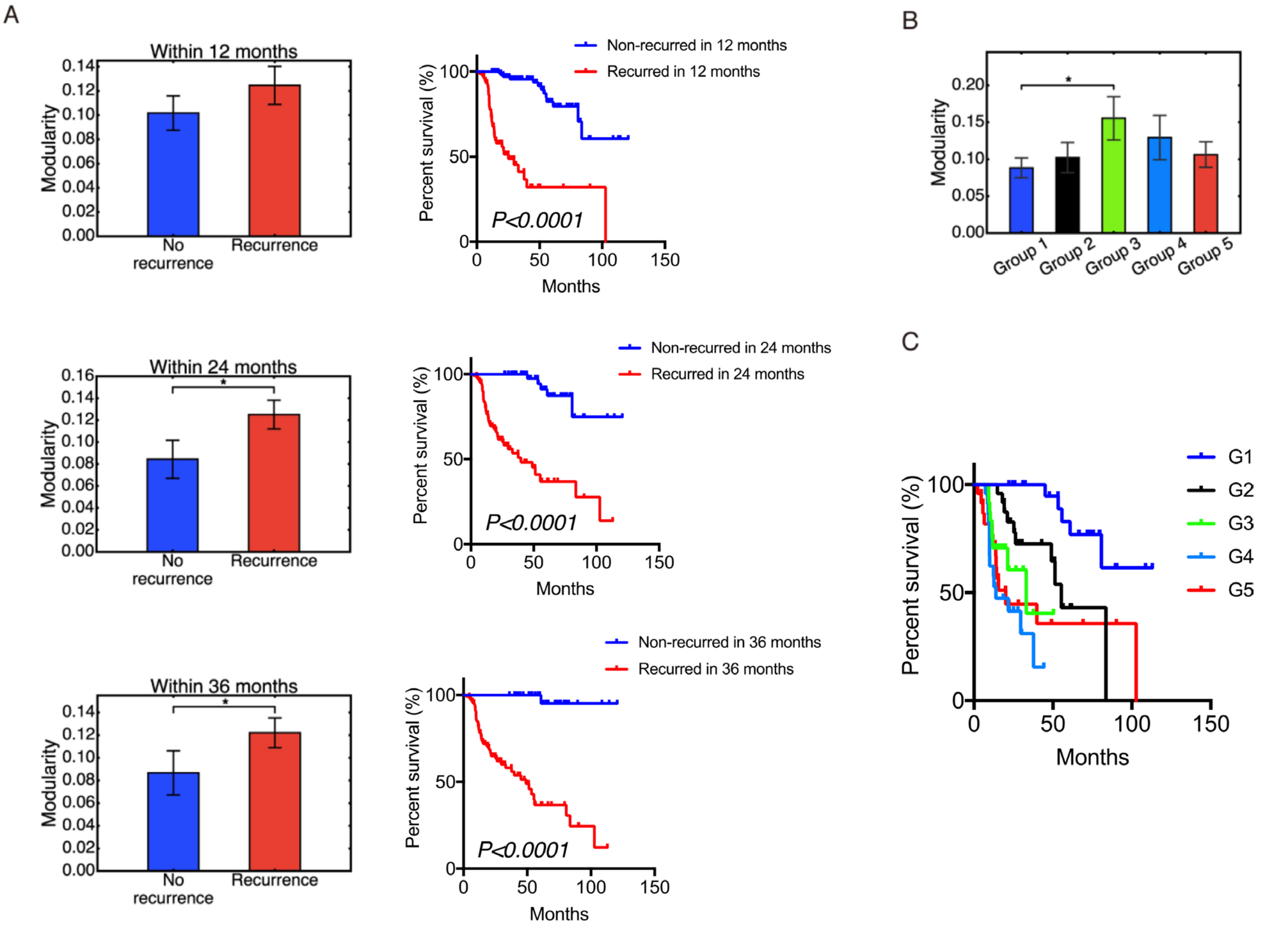

Supplementary Figure 8: Modularity and tumor recurrence for HCC patients with no distant metastasis. (**A**) Modularity (left panels) and Kaplan-Meier overall survival curves (right panels) of HCC patients that were stratified into recurrence and non-recurrence group within 12, 24 and 36 months. (**B**) Non-monotonic change of modularity with tumor recurrence time among patients with recurred tumor. HCC patients in group 1 have the longest recurrence time, and HCC patients in group 5 have the shortest recurrence time. (**C**) Kaplan-Meier overall survival curves of each group. Here, '*' represents $0.01 < p \leq 0.05$. If there is no labeling of the significance level, it means the difference is not significant. Significant *p*-values in (C) are as follows: $p(G1,G2) < 0.01$, $p(G1,G3) < 0.001$, $p(G1,G4) < 0.0001$, $p(G1,G5) < 0.001$, and $p(G2,G4) < 0.001$.
